# The nonlinear relationship between cerebrospinal fluid Aβ_42_ and tau in preclinical Alzheimer’s disease

**DOI:** 10.1371/journal.pone.0191240

**Published:** 2018-02-07

**Authors:** Mony J. de Leon, Elizabeth Pirraglia, Ricardo S. Osorio, Lidia Glodzik, Les Saint-Louis, Hee-Jin Kim, Juan Fortea, Silvia Fossati, Eugene Laska, Carole Siegel, Tracy Butler, Yi Li, Henry Rusinek, Henrik Zetterberg, Kaj Blennow, the Alzheimer’s Disease Neuroimaging Initiative, the National Alzheimer’s Coordinating Center

**Affiliations:** 1 Department of Psychiatry, Center for Brain Health, NYU Medical Center, New York, New York, United States of America; 2 Department of Population Health, Division of Biostatistics, NYU Medical Center, New York, New York, United States of America; 3 Nathan Kline Institute for Psychiatric Research, Orangeburg, New York, United States of America; 4 Department of Radiology, NYU Medical Center, New York, New York, United States of America; 5 Lennox Hill Radiology, New York, New York, United States of America; 6 Department of Neurology, Memory Unit, Hanyang University, Seoul, Korea; 7 Hospital de la Santa Creu i Sant Pau, Universitat Autònoma de Barcelona, Barcelona, Spain; 8 Sahlgrenska University Hospital, University of Gothenburg, Mölndal, Sweden; 9 Institute of Neuroscience and Physiology, Department of Psychiatry and Neurochemistry, the Sahlgrenska Academy at the University of Gothenburg, Mölndal, Sweden; 10 Department of Molecular Neuroscience, UCL Institute of Neurology, London, United Kingdom; 11 UK Dementia Research Institute, London, United Kingdom; Banner Alzheimer's Institute, UNITED STATES

## Abstract

Cerebrospinal fluid (CSF) studies consistently show that CSF levels of amyloid-beta 1–42 (Aβ_42_) are reduced and tau levels increased prior to the onset of cognitive decline related to Alzheimer’s disease (AD). However, the preclinical prediction accuracy for low CSF Aβ_42_ levels, a surrogate for brain Aβ_42_ deposits, is not high. Moreover, the pathology data suggests a course initiated by tauopathy contradicting the contemporary clinical view of an Aβ initiated cascade. CSF Aβ_42_ and tau data from 3 normal aging cohorts (45–90 years) were combined to test both cross-sectional (n = 766) and longitudinal (n = 651) hypotheses: 1) that the relationship between CSF levels of Aβ_42_ and tau are not linear over the adult life-span; and 2) that non-linear models improve the prediction of cognitive decline. Supporting the hypotheses, the results showed that a u-shaped quadratic fit (Aβ^2^) best describes the relationship for CSF Aβ_42_ with CSF tau levels. Furthermore we found that the relationship between Aβ_42_ and tau changes with age—between 45 and 70 years there is a positive linear association, whereas between 71 and 90 years there is a negative linear association between Aβ_42_ and tau. The quadratic effect appears to be unique to Aβ_42_, as Aβ_38_ and Aβ_40_ showed only positive linear relationships with age and CSF tau. Importantly, we observed the prediction of cognitive decline was improved by considering both high and low levels of Aβ_42._ Overall, these data suggest an earlier preclinical stage than currently appreciated, marked by CSF elevations in tau and accompanied by either elevations or reductions in Aβ_42_. Future studies are needed to examine potential mechanisms such as failing CSF clearance as a common factor elevating CSF Aβ_xx_ analyte levels prior to Aβ_42_ deposition in brain.

## Introduction

Improved biomarker characterization during the presymptomatic stages of Alzheimer’s disease (AD) is necessary to adequately assess the risk for cognitive decline and optimize interventions. It is widely believed that in elderly at risk for AD, reductions in the cerebrospinal fluid (CSF) levels of amyloid beta 1–42 (Aβ_42_), which are associated with brain amyloid deposition [1,2], precede elevations in CSF tau levels, a marker of neurodegeneration [3]. Support for this sequence of CSF biomarker changes comes from preclinical studies showing that lower CSF Aβ_42_ levels predict cognitive decline [4,5]. Others report that CSF Aβ_42_ and tau levels configured as a ratio, are superior to univariate predictors of future impairment [1], thus further highlighting the value of CSF Aβ_42_ reductions. However, this clinical view of early AD lesions conflicts with the neuropathology which identifies tauopathy more commonly than Aβ lesions in younger brains [6]. Moreover, adding to the uncertainty, normal aging studies have been inconsistent, showing that CSF Aβ_42_ levels increase [7,8] or decrease [9], or do not change with age [10]. Offering a clue to this discrepancy, transgenic animal models show CSF Aβ_42_ elevations occur prior to Aβ_42_ reductions and brain deposition [11], a trend also seen in early onset AD [12,13]. We reasoned that these divergent human aging findings could be explained by under sampling a non-linear Aβ_42_ trajectory with the added complexity of non-standardized cohorts. Utilizing cross-sectional (n = 766) and longitudinal data (n = 651) from three normal elderly cohorts with similar clinical and CSF protocols, we tested and confirm two major hypotheses: 1) CSF Aβ_42_ levels have a non-linear relationship with both age and CSF biomarkers for tau pathology. We observed during mid to late-adult life, a quadratic or U-function uniquely and significantly described the CSF Aβ42 relationships to age and to CSF tau levels. For CSF Aβ38 or Aβ40, only linear relationships were found with age and tau levels; and 2) the cross-sectional and longitudinal results confirm the hypothesis that elevated Aβ42 levels contribute to the prediction of future cognitive decline.

## Methods

### Human subjects

**Fig 1:**
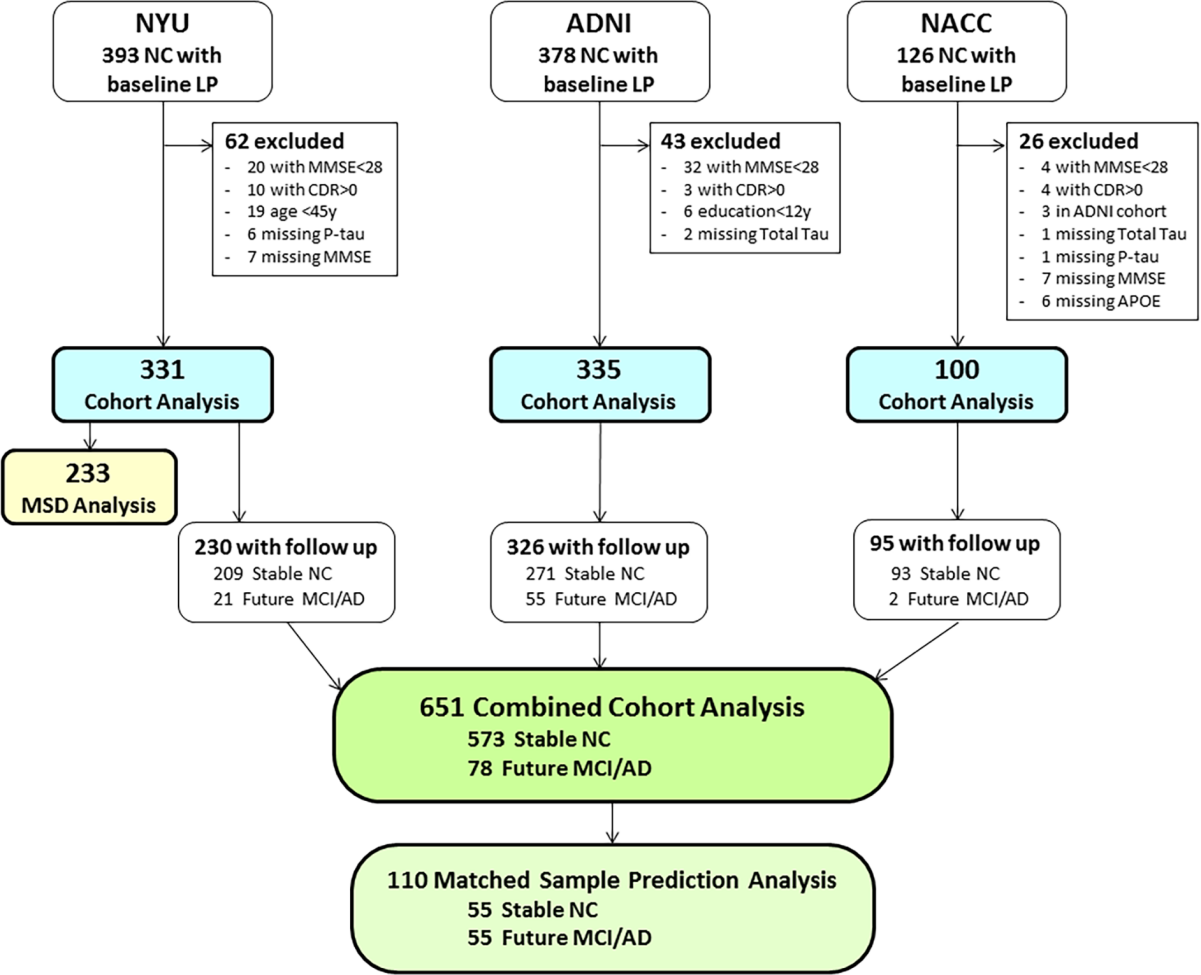
Subject flow chart. The patient flow chart shows the inclusion and exclusions of subjects in the analyses conducted for this study.

The data were derived from three prospective and currently active longitudinal studies that sample aging subjects with normal cognition (NC). Written informed consent was obtained from all subjects. The NYU studies were approved by the NYU School of Medicine Institutional Review Board (IRB) and the ADNI and NACC studies were IRB approved by each of collaborating sites. All of the reported studies and all clinical investigations were conducted according to the principles expressed in the Declaration of Helsinki. The authors were not involved in data collection for the ADNI and NACC studies and both datasets were anonymized prior to being received by the authors. In total there were 766 subjects with LP data in the individual cohort studies. Of those, 651 subjects had a clinical follow-up and were included in the combined cross-sectional and prediction studies of cognitive outcome. For the prediction study, 573 subjects retained the clinical diagnosis of normal (Stable NC) at their last clinical follow-up evaluation and 78 demonstrated future cognitive decline consistent with mild cognitive impairment (MCI) or AD (Fig 1). A second prediction analysis was done with a subset of the Stable NC and Future MCI/AD groups matched 1:1. The matched subjects were selected by having an exact match of a Stable NC subject to a Future MCI/AD subject in terms of cohort, sex, ApoE4 status, race (Caucasion or non-Caucasian), baseline age split at 75y (<75y or >75y); as well as matching age within 5 years, years of education within 2 years, and follow-up time within 2 years. Exact sampling was done without replacement, and for 23 decliners no available matching control was found. Consequently, we examined 55 Future MCI/AD and 55 matching Stable NC.

The NYU cohort of community dwelling volunteers (n = 331), included n = 230 with a clinical follow-up. These data were derived from NIH supported RO1 aging studies between 1997 and 2016 (MdeL, Principal Investigator). All subjects received a standard protocol consisting of medical, neurological, psychiatric, neuropsychological testing, clinical laboratory, magnetic resonance imaging (MRI) examinations, and lumbar puncture (LP). The primary goal of the NYU studies was to examine CSF biomarkers and magnetic resonance imaging (MRI) predictors of cognitive impairment in aging.

The second cohort (n = 335, including n = 326 with a clinical follow-up) was enrolled in the Alzheimer’s Disease Neuroimaging Initiative (ADNI) database (adni.loni.usc.edu). The ADNI was launched in 2003 as a public-private partnership, led by Principal Investigator Michael W. Weiner, MD. The primary goal of ADNI has been to use serial magnetic resonance imaging (MRI), positron emission tomography (PET), fluid biological markers, and clinical and neuropsychological assessment to describe and predict progressive cognitive impairment as related to Alzheimer’s disease (AD). For up-to-date information, see www.adni-info.org.

The third cohort (n = 100, including n = 95 with a clinical follow-up) was derived from the National Alzheimer’s Coordinating Center (NACC). NACC was established by the NIH National Institute on Aging (NIA) in 1999 to facilitate collaborative research across Alzheimer’s Disease Centers (ADC). NACC has developed and maintains a large relational database of standardized clinical and neuropathological research data in partnership with the Alzheimer's Disease Genetics Consortium and the National Cell Repository for Alzheimer's Disease. Of four participating NACC sites, data from only one site was selected. One dropped site had only two cognitively normal subjects and two dropped sites did not provide batch adjustments for the CSF values. Further, to avoid any possible overlap, three NACC subjects from the selected site that matched the ADNI data by year and month of birth, sex, education and APOE ε4 carrier status, were removed from the NACC data set. The data used was from CSF collected between January 2010 and February 2013.

### Study enrollment criteria

All included subjects were clinically evaluated as having normal cognition (NC) and were between the ages 45 to 90y at the time of the baseline LP. All subjects had 12 or more years of education, *APOE* genotyping, a Mini-Mental State Examination (MMSE) [14,14] score of 28 or greater, a Clinical Dementia Rating (CDR) [15] score of 0 and/or a Global Deterioration Scale (GDS) [4] score of 1 or 2. In total, 131 subjects were excluded—30 subjects were dropped due to missing data and an additional 101 subjects did not meet the above screening characteristics despite having a diagnosis of normal cognition.

### Lumbar puncture, CSF collection and assays

The NYU procedures for the lumbar puncture and CSF handling are published [16] and are consistent with the recommendations of Vanderstichle et al [17]. In brief, CSF Amyloid beta (Aβ_42_) levels and Total tau (T-tau) were measured using a standard ELISA protocol (Innotest®, Innogenetics, Ghent, Belgium). An Innogenetics sandwich ELISA assay was used to detect tau phosphorylated at threonine 181 (P-tau181). In this assay a phospho-dependent capture antibody, AT270 (P176PAPKTpP132), and a human specific tau detection antibody, HT7 (P159PGQK163), were utilized [18]. Batch wise rescaling of Aβ_42_ was performed using linear regression with a single reference batch of 236 NC subjects. For a subset of 233 NYU subjects, the Aβ_42,_ Aβ_38,_ and Aβ_40_ levels were additionally examined with the MSD Abeta Triplex (Meso Scale Discovery, Rockville, Maryland). All assays were conducted at the Sahlgrenska University Hospital in Sweden by board-certified laboratory technicians who were blinded to clinical data.

The procedures used by ADNI for assay technology, validation, quality control and adjustments for batch variations are found online in the ADNI data primer for CSF analyses (http://adni.info.org/). Briefly, the CSF AD biomarkers Aβ_42,_ P-tau181 and T-tau were measured on the multiplex xMAP Luminex platform (Luminex Corp, Austin, TX) with Innogenetics immunoassay kit–based reagents (INNO-BIA AlzBio3, Ghent, Belgium [19]). The biomarker data file (UPENNBIOMK_MASTER.csv) was downloaded from the ADNI website on January 2017.

Unlike NYU and ADNI, the published NACC reports do not state CSF collection methods. However, the included NACC site used an ELISA analytic protocol similar to the method used at NYU with all provided data assayed in one batch. When referring to P-tau181 and T-tau collectively, we use the term X-tau; and when referring to multiple Aβ fragments we use Aβ_xx_.

### Statistical analyses

Demographic differences between cohorts and Outcome Group were assessed using ANOVA with Tukey post hoc correction for pairwise comparisons for continuous variables. Chi-square tests with Bonferroni correction for pairwise comparisons were used to test nominal variables for group differences. The standard confounds of age, sex and ApoE4 status were added to all statistical models as covariates with the exception of models with demographically matched outcome groups.

We observed in the individual cohort scatterplots that both high and low levels Aβ_42_ were associated high Tau levels. From this observation we hypothesized that a quadratic fit of Aβ_42_ would explain more of the variance in the Tau measures than the linear fit of Aβ_42_ alone. Linear regression models, with the T-Tau and P-Tau181 measures as dependent variables, were used to test this hypothesis. The linear Aβ_42_ term followed by the quadratic Aβ_42_ term (Aβ^2^) were added consecutively to test the incremental change in the model with the standard confounds. To account for possible heteroscedasticity of the estimators, robust (Huber-White) sandwich estimators were used to estimate the standard errors. Sensitivity analysis was performed using all available subjects with normal cognition at their baseline LP (no exclusions) from each cohort.

To have sufficient power to investigate possible interaction effects and the relationship of Aβ^2^ with future cognitive decline, subjects with follow-up clinical data from the three cohorts were combined and analyzed using Generalized Estimating Equations (GEE). The GEE allowed us to account for possible clustering of subjects within cohorts. We assumed an unstructured correlation matrix in all GEE models with cohort included as a within-subjects effect. The GEE models were analyzed first on the raw biomarker values. However, since the cohorts used different assays, the scale of the raw values were different for each cohort making graphing and the interpretation of model coefficients problematic. For this reason, z scores for each cohort were calculated using the mean and standard deviation from the raw biomarker values. The results are reported and graphed using the z scores.

The X-Tau measures were examined as the dependent variables with the identity link function in the GEE, with the standard covariates, Aβ_42_, Aβ^2^, and the interactions of the standard confounds with Aβ_42_ and Aβ^2^. In the presence of significant interaction terms, the data was subdivided and reanalyzed to explore the interaction.

We hypothesized that both high and low Aβ_42_ were associated with future cognitive decline and that this effect would be stronger in younger subjects. To test this hypothesis we used the GEE with the logit link and Outcome group as a binary dependent variable. Aβ_42_ was added to the standard confounds as an independent variable to test a linear association with Future MCI/AD_._ Subsequently, we added Aβ^2^ and its interaction with age. Observing a significant interaction with age, the sample was divided at the median age of the Future MCI/AD group (75y) and the GEE models including the standard confounds, Aβ_42_ and Aβ^2^ were evaluated separately in young (45-75y) and old age groups (75.1-86y). This analysis was replicated in the matched outcome groups.

In the subset of 233 NYU subjects who additionally had Aβ_38,_ Aβ_40,_ and Aβ_42_ measured with MSD, we hypothesized that Aβ_42_ would show quadratic relationships with X-Tau measures, whereas Aβ_38_ and Aβ_40_ would show linear relationships. To test this hypothesis we used linear regression models with the X-Tau measures as the dependent variables and the linear and quadratic terms for Aβ_38,_ Aβ_40_ and Aβ_42_ as independent variables.

All analyses were checked for violations of the model assumptions and any conflicts are reported. The Box Cox transformation procedure [20] was used to determine the most appropriate power transformation to reconfigure values to a normal distribution. Differences in variances were tested using Levene's Test for Equality of Variances. All variables were centered for the calculation of higher order terms to avoid multicollinearity with the main effects. For all results, statistical significance was defined as a two-sided p-value of less than 5%. Statistical analyses were performed using IBM SPSS (Version 23.0) and figures were rendered in Adobe Illustrator (CC 2015).

## Results

### Between cohort comparisons

The three cohorts did not differ in education level, MMSE, or proportions of *APOE* ε4 carriers. The cohorts differed in age (F = 148.2, p < .01) and in the proportions of Caucasian (χ^2^ = 10.7, p < .01), and female subjects (χ^2^ = 14.2, p < .01), see Table 1.

**Table 1:** Cross sectional study demographics and descriptive variables by cohort.

	NYU	ADNI	NACC
**N**	331	335	100
**Age ^**a**^**	64.7 ± 9.1 (45-88) ^b^^,^^c^	73.7 ± 5.8 (56-90) ^b^^,^^d^	61.9 ± 8.9 (45-83) ^c^^,^^d^
**Education ^**a**^**	16.8 ± 2.1 (12-20)	16.7 ± 2.4 (12-20)	16.4 ± 2.3 (12-20)
**% Female**	65% ^c^	54% ^b^^,^^d^	71% ^c^
**% Caucasian**	92% ^b^	90% ^b^	100% ^c^^,^^d^
**% E4+**	30%	28%	36%
**MMSE ^**a**^**	29.5 ± 0.7 (28-30)	± 0.7 (28-30)	29.4 ± 0.7 (28-30)

a. Values are mean ± standard deviation (range).

b. Statistically significant difference from NACC cohort (p < .05).

c. Statistically significant difference from ADNI cohort (p < .05).

d. Statistically significant difference from NYU cohort (p < .05).

The X-tau and Aβ_42_ measures were not normally distributed in any of the cohorts. The X-tau values were positively skewed and the natural log transformation effectively normalized the distributions in all three cohorts. Aβ_42_ was platykurtic with a high degree of negative excess kurtosis (NYU: kurtosis = -.54; ADNI: kurtosis = -.99; NACC: kurtosis = -.32). There was no evident power transformation that normalized the Aβ_42_ distributions.

### Cross-sectional CSF biomarker relationships in three cohorts

**Fig 2:**
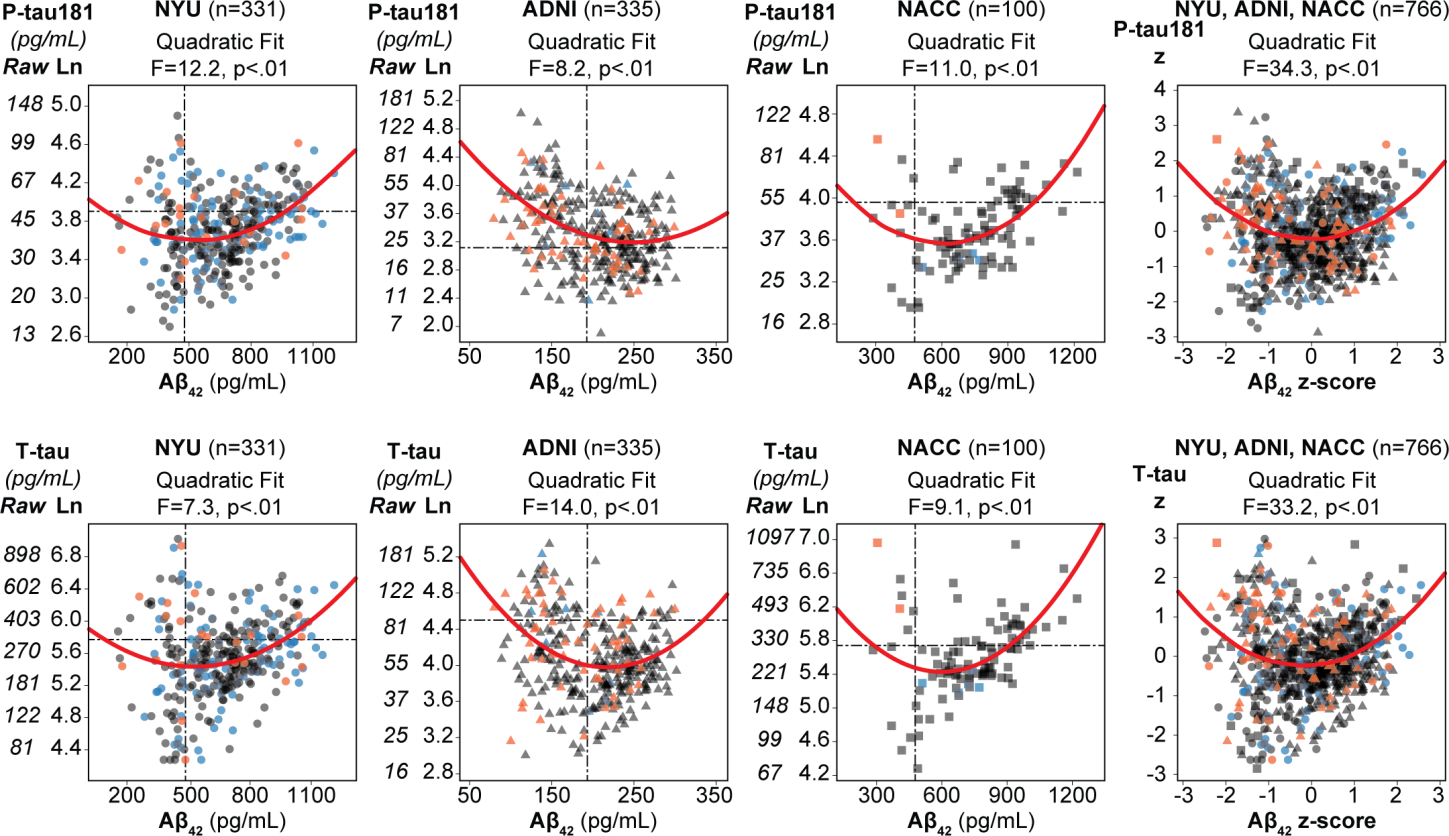
Relationship between Aβ_42_ and tau in 3 cohorts. Scatter plots for each cohort with Aβ42 on the x-axis and Tau with the natural log transformation (and the associated raw values) on the y-axis. Individual subjects are shown as circles for NYU, triangles for ADNI and squares for NACC. The outcome groups are indicated by color with the cross-sectional NL in blue, Stable NC in gray, and Future MCI/AD in orange. The quadratic fit is shown as a solid red line. The F statistic is from additive value of the quadratic fit to the model including the linear fit and standard confounds. Published cut offs for each biomarker [19,21] are depicted with dashed lines. The cohorts were combined after Z scoring.

The scatter plots of Aβ_42_ with the X-tau show in each cohort that Aβ_42_ bifurcates as the X-tau measures increase, with high and low values of Aβ_42_ being associated with high X-Tau values (Fig 2). In all three cohorts, the Aβ^2^ term (quadratic), was a significant addition to the linear Aβ_42_ models predicting either P-tau181 or T-tau. Specifically, this was found for the prediction of P-tau181 (NYU: R^2^ change = 3%, F change = 12.2, p < .01; ADNI: R^2^ change = 2%, F change = 8.2, p < .01; NACC: R^2^ change = 8%, F change = 11.0, p < .01) and for the prediction of T-tau (NYU: R^2^ change = 2%, F change = 7.3, p < .01; ADNI: R^2^ change = 4%, F change = 14.0, p < .01; NACC: R^2^ change = 6%, F change = 9.1, p < .01; Table 2). The data also showed that Aβ_42_ was not suitable as a dependent variable because all cohorts showed heteroscedasticity in the variances of the residuals in the models with Aβ_42_ as the dependent variable. The results in all three cohorts were confirmed with sensitivity analyses including all normal subjects.

**Table 2:** Linear regression models predicting X-tau (log transformed) by cohort.

**Dependent variable**		**P-tau 181**											
**Model** ^**a**^	**Independent Variables**	**NYU** (n = 331)				**ADNI** (n = 335)				**NACC** (n = 100)			
		**AIC**	**β**^**b**^	**95% CI**^**c**^	**p**	**AIC**	**β**^**b**^	**95% CI**^**c**^	**p**	**AIC**	**β**^**b**^	**95% CI**^**c**^	**p**
**Model 1**	Age ^d^	275	0.25	[.15,.34]	< .01	466	0.13	[.03, .22]	< .01	64	0.35	[.16, .54]	< .01
	Sex		0.04	[-.06, .14]	0.42		0.04	[-.06, .14]	0.45		-0.01	[-.19, .16]	0.87
	ApoE4		0.07	[-.04, .17]	0.22		0.30	[.19, .40]	< .01		0.07	[-.11, .26]	0.44
**Model 2**	model 1+ Aβ_42_ linear	247^e^	0.28	[.18,.38]	< .01	445^d^	-0.27	[-.38, -.17]	< .01	54^d^	0.33	[.11, .55]	< .01
**Model 3**	model 2 + Aβ_42_ quadratic	236^e^	0.13	[.06, .21]	< .01	439^d^	0.15	[.05, .25]	< .01	45^d^	0.25	[.09, .41]	< .01
**Dependent variable**		**T-tau**											
**Model** ^**a**^	**Independent Variables**	**NYU** (n = 331)				**ADNI** (n = 335)				**NACC** (n = 100)			
		**AIC**	**β**^**b**^	**95% CI**	**p**	**AIC**	**β**^**b**^	**95% CI**	**p**	**AIC**	**β**^**b**^	**95% CI**	**p**
**Model 1**	Age ^d^	406	0.36	[.27, .46]	< .01	382	0.25	[.15, .36]	< .01	121	0.43	[.25, .61]	< .01
	Sex		0.02	[-.09, .12]	0.77		0.08	[-.02, .19]	0.12		0.00	[-.18, .19]	0.98
	ApoE4		0.12	[.02, .23]	< .05		0.20	[.09, .32]	< .01		0.07	[-.11, .25]	0.45
**Model 2**	model 1+ Aβ_42_ linear	376^e^	0.28	[.18, .38]	< .01	382	-0.09	[-.20, .03]	0.14	110^d^	0.32	[.09, .56]	< .01
**Model 3**	model 2 + Aβ_42_ quadratic	371^e^	0.10	[.04, .17]	< .01	370^d^	0.20	[.09, .31]	< .01	103^d^	0.22	[.05, .39]	< .05

a. All models are cumulative with respect to the variables included in the prior models.

b. Standardized β coefficients give a consistent scale of the slope across variables with 1 unit = 1 standard deviation for each variable.

c. The 95% CI of the β coefficients are calulated using robust (Huber-White) sandwich estimators of the standard error.

d. Values are in years.

e. Significant likelihood ratio test compared to previous model.

### The combined sample: Relationships between CSF biomarkers

The quadratic Aβ_42_ term predicting the X-tau measures was also significant in the combined sample, n = 651: (P-tau181: β = 0.19, 95%CI: 0.12 to 0.26, p < .01; T-tau: β = 0.18, 95%CI: 0.12 to 0.24, p < .01). No significant interactions were found between the quadratic Aβ_42_ term and the standard confounds, indicating that the strength of the quadratic association did not change with age, between ApoE4 carriers and non-carriers, nor between males and females.

**Fig 3:**
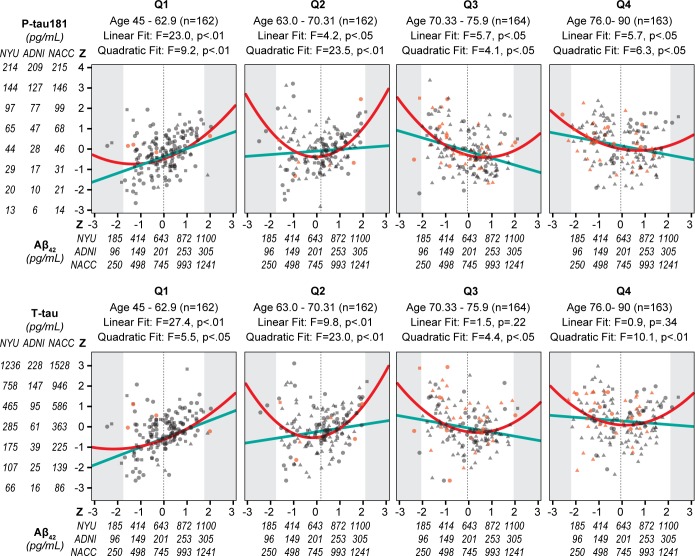
Relationship between age quartiles, Aβ_42_ and X-tau in 3 cohorts combined. Scatter plots by age quartiles with the x-axis showing for Aβ_42_ the combined z scores and raw values by cohort. The y-axis shows the log transformed P-tau181 and T-tau raw scores by cohort and the combined z scores. Individual subjects are shown as circles for NYU, triangles for ADNI and squares for NACC. The outcome groups are indicated by color with the cross-sectional NL in blue, Stable NC in gray, and Future MCI/AD in orange. For each quartile, the linear fit is shown as a solid light blue line and the quadratic fit as a solid red line. The shaded area represents the area outside the 95% CI of the Aβ_42_ values for each quartile and the vertical dotted line is at the mean Aβ_42_ for each quartile.

However, for the linear Aβ_42_ term significant interactions with age were found: (P-tau181: β = -0.025, 95%CI: -0.033 to -0.016, p < .01; T-tau: β = -0.019, 95%CI: -0.028 to -0.010, p < .01). To investigate the interaction of age on the relationship between Aβ_42_ and X-tau, we broke the combined sample into age quartiles. The younger two quartiles showed positive linear correlations between Aβ_42_ and the X-tau biomarkers (Fig 3 and Table 3). Whereas in the two older quartiles, Aβ_42_ was negatively associated with P-tau181, but the negative association with T-tau did not reach significance (Fig 3). Interactions of Aβ_42_ with sex and ApoE4 status failed to reach statistical significance.

**Table 3:** Aβ_42_ in the prediction of X-tau by age quartiles.

Quartile	N	age range	Aβ_42_ variance^a^	Dependent variable	linear fit Aβ_42_			quadratic fit Aβ_42_		
					β ^b^	95% CI ^c^	p value	β ^b^	95% CI ^c^	p value
**Q1**	162	45.1–62.9	0.80	P-tau181	0.42	0.25, 0.59	< .01	0.18	0.06, 0.30	< .01
**Q2**	162	63.0–70.31	0.96		0.20	0.01, 0.39	< .05	0.28	0.17, 0.39	< .01
**Q3**	164	70.33–75.9	1.03		-0.23	-0.41, -0.04	< .05	0.19	0.01, 0.36	< .05
**Q4**	163	76.0–89.7	1.18		-0.18	-0.32, -0.03	< .05	0.14	0.03, 0.25	< .05
**Q1**	same as above			T-tau	0.44	0.27, 0.60	< .01	0.14	0.02, 0.25	< .05
**Q2**					0.30	0.11, 0.49	< .01	0.25	0.15,0.35	< .01
**Q3**					-0.11	-0.29, 0.07	0.22	0.17	0.01, 0.33	< .05
**Q4**					-0.07	-0.22, 0.08	0.34	0.18	0.07, 0.29	< .01

a. Significant increase in variance across quartiles.

b. β coefficients are unstandardized values from the GEE with CSF z scores.

c. The 95% CI of the β coefficients are calulated using robust (Huber-White) sandwich estimators of the standard error.

### Increase in the variance of Aβ_42_ with age

Interestingly, the variance of Aβ_42_ significantly increased across the age quartiles (Levene's Test of Equality of Error Variances F = 5.1, p < .01), despite the absence of significant differences in the means between the age quartiles (Fig 3). In other words, with increasing age there are increased numbers of subjects with high and low CSF Aβ_42_ levels. Since older age, low CSF Aβ_42_, and high tau levels are associated with a greater risk of AD, these changes in the variance of Aβ_42_ suggested that both high and low Aβ_42_ values could be associated with future decline. This hypothesis was tested in the GEE model predicting Outcome group.

### Outcome group descriptions

The association of the CSF biomarkers and future clinical decline was examined in the combined sample of 78 Future MCI/AD and 573 Stable NC, (Table 4). Demographically, the Future MCI/AD were older (t = 7.5, p < .01), had less education (t = -2.5, p < .05), and a higher proportion of males (χ^2^ = 4.6, p < .05). By design, there were no differences in the matched group in terms of the demographic variables.

**Table 4:** Demographics and baseline descriptive variables for outcome groups.

	Stable NC	Future MCI/AD	Macthed sample age 51-75y		Matched sample age 75-86y	
			Stable NC^b^	Future MCI/AD^b^	Stable NC^b^	Future MCI/AD^b^
n (NYU,ADNI,NACC)	573 (209,271,93)	78 (21,55,2)	30 (11,19,0)	30 (11,19,0)	25 (4,21,0)	25 (4,21,0)
Age ^a^	68.2 ± 8.9 (45–90)	74.5 ± 6.7 (51–86)^c^	71.0 ± 4.2 (55.3–75.0)	69.7 ± 5.4 (50.9–75.0)	81.2 ± 3.2 (75.1–86.3)	79.6 ± 2.9 (75.1–85.8)
Education ^a^	16.8 ± 2.2 (12–20)	16.1 ± 2.4 (12–20) ^c^	16.7 ± 1.7 (12–20)	16.9 ± 1.9 (12–20)	16.3 ± 2.4 (12–20)	16.0 ± 2.6 (12–20)
% Female	61%	49% ^c^	50%	50%	40%	40%
% Caucasian	94%	90%	90%	90%	100%	100%
% E4+	30%	31%	23%	23%	16%	16%
MMSE ^a^	29.4 ± 0.7 (28–30)	29.2 ± 0.8 (28–30)	29.3 ± 0.7 (28–30)	29.3 ± 0.7 (28–30)	29.4 ± 0.8 (28–30)	29.1 ± 0.8 (28–30)
Project follow-up time ^a^	3.6 ± 2.4 (0.5–15.3)	4.4 ± 2.8 (0.5–10.2)	4.0 ± 2.6 (0.9–10.1)	4.1 ± 2.3 (1.2–9.0)	3.7 ± 2.4 (0.5–9.2)	3.6 ± 2.6 (0.5–10.1)

a. Values are mean ± standard deviation (range).

b. A subset of the Stable NC and Future MCI/AD were matched 1:1 on demographic variables.

c. Statistically significant difference from Stable NC (p < .05).

### Aβ_42_ and Aβ^2^ associations with clinical outcome

Our combined sample findings replicate prior observations of lower levels of Aβ_42_ being associated with Future MCI/AD (Wald Chi-square = 7.3, OR = 1.4, p < .01). However, the addition of the quadratic (Aβ^2^) term and it’s interaction with age (Wald Chi-square = 3.9, OR = 1.03, p < .05) confirmed the hypothesis that both high and low Aβ_42_ values contribute to the prediction of future clinical decline particularly at a younger age. Splitting the full sample at the median age of the decline group (75y) and fixing the specificity at 75%, we found that in the younger group (stable NC n = 434 vs. Future MCI/AD n = 39) the quadratic Aβ_42_ has the greater sensitivity (72%) compared to the linear Aβ_42_ term (sensitivity = 61%). In the >75y sample (stable NC n = 139 vs. Future MCI/AD n = 39), neither the quadratic nor the linear Aβ_42_ term demonstrated sensitivity (31% and 33% respectively).

In the matched sample study the linear Aβ_42_ term did not reach significance. However, the quadratic (Aβ^2^) term was a significant predictor of outcome group (Wald Chi square = 5.9, OR = 1.6, p < .05). This result was driven by the strong quadratic effect in the younger (<75y) sample (Wald Chi-square = 4.2, OR = 1.6, p < .05). Moreover, like the larger unmatched sample, the quadratic (Aβ^2^) term did not reach significance in the older (>75y) subjects.

### MSD analyses: The relationships between Aβ_42,_ Aβ_38,_ and Aβ_40_ with age and X-tau

As the relationship between Aβ_42_ to age and to X-tau can be affected by brain Aβ deposition, we also examined Aβ_38_ and Aβ_40_ which like Aβ_42_ are sensitive to production but unlike Aβ_42,_ are known to have minimal brain accumulations [22]. We observed that Aβ_38_ and Aβ_40_ had strong positive linear relationships with age: (R^2^ change = 5%, F change = 11.0, p < .01 and R^2^ change = 5%, F change = 13.2, p < .01, respectively), whereas Aβ_42_ did not show a significant linear age effect (replicating the finding for the total sample).

**Fig 4:**
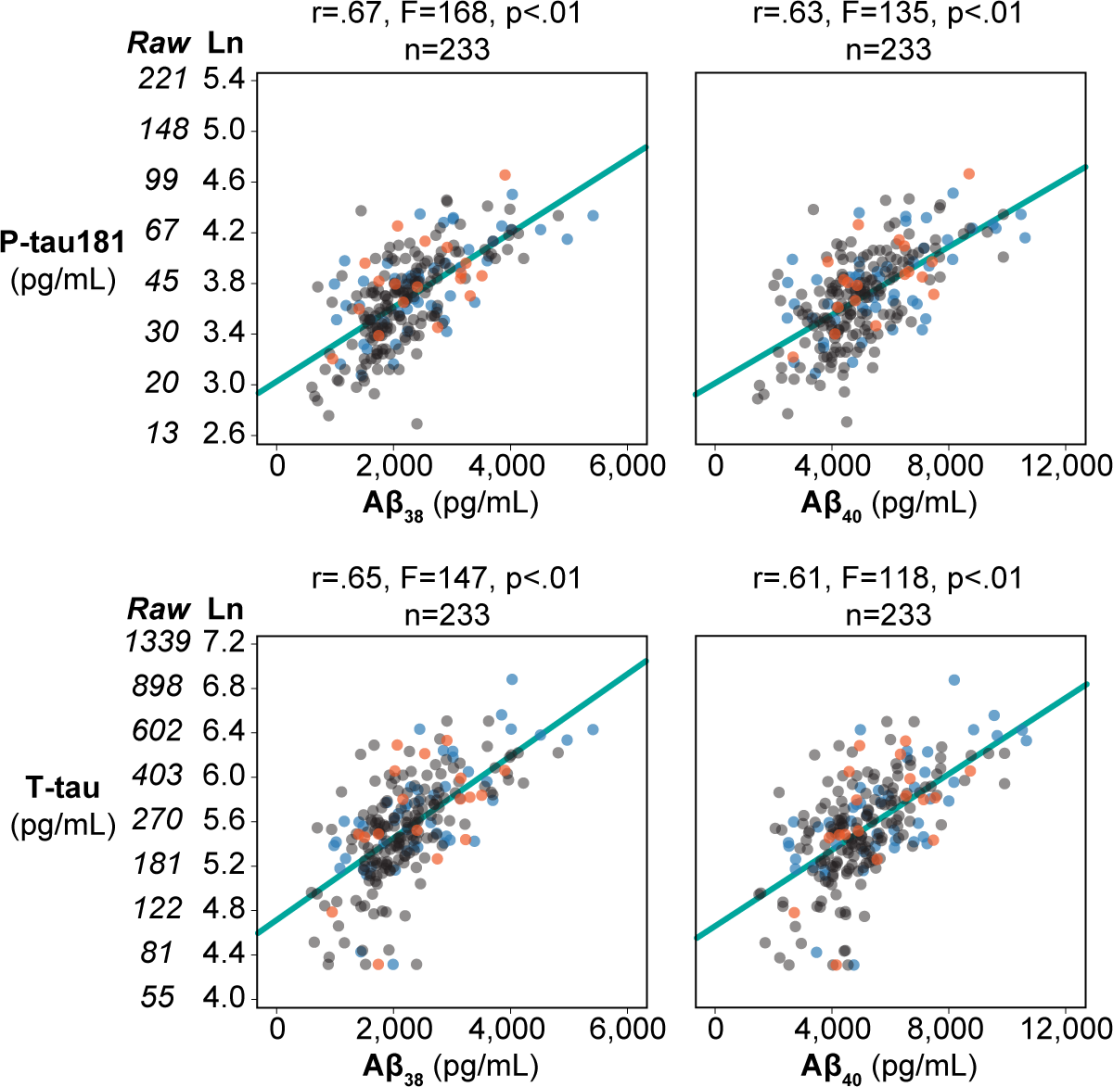
Relationship of X-tau with Aβ_38_ and Aβ_40_. Scatter plots for n = 233 depicting Aβ_38_ and Aβ_40_ on the x-axis and the natural log transformation of X-Tau (and the associated raw values) on the y-axis. The outcome groups are indicated by color with the cross-sectional NL in blue, Stable NC in gray, and Future MCI/AD in orange. The linear fit is shown as a solid light blue line.

Both Aβ_38_ and Aβ_40_ show strong positive linear effects with X-tau. This is clearly seen for T-tau, (R^2^ change = 35%, F change = 147, p < .01, and R^2^ change = 31%, F change = 118, p < .01, respectively), as well as for P-tau181, (R^2^ change = 40%, F change = 168, p < .01, and R^2^ change = 35%, F change = 135, p < .01, respectively, Fig 4). As in the total sample, Aβ^2^ uniquely shows the quadratic relationship to P-tau181 (R^2^ change = 2%, F change = 4.2, p < .05) and to T-tau (R^2^ change = 2%, F change = 4.6, p < .05).

## Discussion

The present study of cognitively normal elderly describes for the first time, quadratic relationships between Aβ_42_ and both P-tau181 and T-tau. We observed a U-function, such that both higher and lower levels of Aβ_42_ were associated with increasing X-tau levels. This effect was observed in three independent subject cohorts and for each age quartile ranging from 45–90 years. Only linear effects were found for either Aβ_38_ or Aβ_40_ with respect to X-Tau levels. Most importantly, we observed the quadratic Aβ_42_ term (Aβ^2^) was superior to the linear Aβ_42_ term as a predictor of future clinical decline, an effect most pronounced in younger subjects. Three important observations were made in this study, all centered on the non-linearity of the Aβ_42_ level.

First, both elevations and reductions in the CSF Aβ_42_ level are associated with increased CSF levels of P-tau181 and T-tau, known biomarkers markers of brain degeneration. These data suggest that Braak’s observation that in aging, tau pathology precedes Aβ_42_ pathology [6], may be under appreciated. CSF biomarker studies that rely on decreased CSF Aβ_42_ levels to identify brain amyloid sequestration and mark the onset of preclinical AD [23] will have missed an earlier stage of elevated Aβ_42_ also accompanied by elevated tau levels. Our data suggest that the use of ratio approaches to identify subjects at risk for AD are not optimal as individuals with higher Aβ_42_ levels would not be identified as at increased risk. Specifically, we observed that in subjects under 75y, adding the quadratic term to the prediction model significantly increased the model from an accuracy of 61% to 72%. For subjects older than 75y, neither the linear nor the quadratic terms predicted outcome.

Of the 78 the Future MCI/AD group, there were 5 subjects (3 from ADNI and 2 from NYU) with a non-AD diagnosis (2 with Parkinson’s, 2 with cortical basal degeneration and 1 with undetermined etiology) at their last visit (1.3 to 6 years after baseline). Removal of these subjects did not affect the quadratic prediction model, and further, none of these 5 subjects had extreme Aβ_42_ values. Rather, their z scores ranged from -1.6 to 1.1 and their mean (z = -.43) did not differ from the mean of the rest of the Future MCI/AD group (z = -.29). We therefore conclude that the quadratic Aβ_42_ measure is a predictor of MCI/AD outcome.

Further, the impact of ignoring the quadratic relationship could also be compounded in a longitudinal design where normal appearing levels may follow elevations. Should CSF Aβ_42_ levels first rise and subsequently fall in the preclinical stages of AD, a subject could have presumably normal appearing Aβ_42_ levels twice, complicating diagnostic examinations and the assessment of Aβ_42_ positivity often used in clinical prevention trials. Temporal evidence in support of the view that Aβ_42_ elevations precede brain Aβ_42_ depositions comes from murine models for AD [11]. To a very limited extent, trends exist in the presenile dementias [12,13].

**Fig 5:**
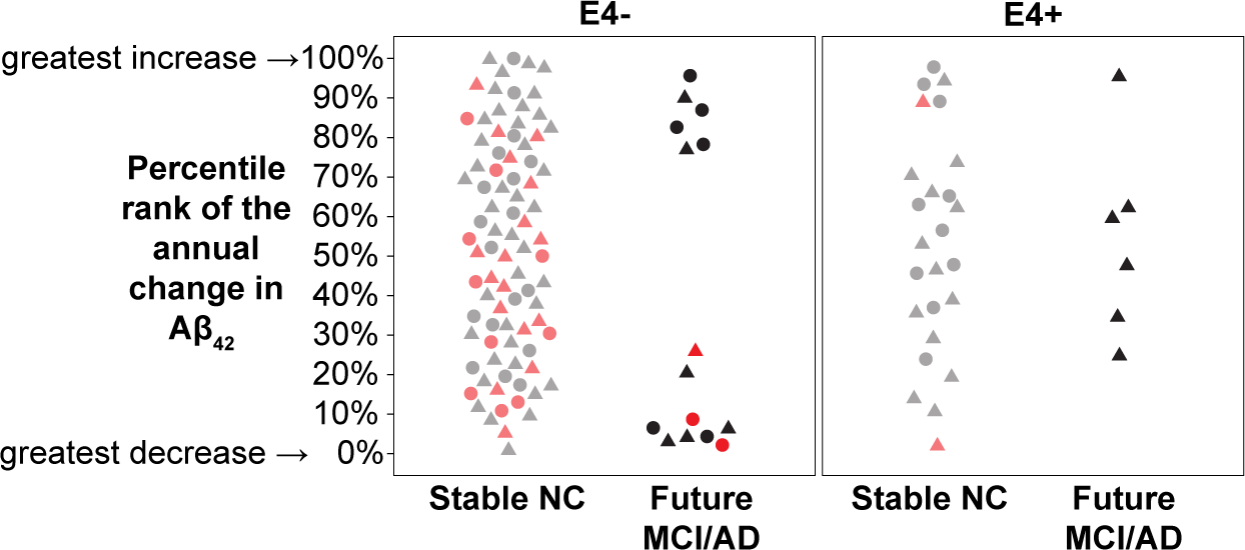
Percentile rank of the annual percent change in Aβ_42_ (ε4 genotype and outcome group). The percentile rankings (by cohort) of the annual percent change in Aβ_42_ are shown on the y-axis. The subjects are split by E4 carrier status on the x-axis then by Outcome group which is indicated with gray markers for stable NC with baseline Aβ_42_ levels below the 75^th^ percentile and light red for those above and black for Future MCI/AD with baseline Aβ_42_ levels below the 75^th^ percentile and red for those above. NYU subjects are represented as circles and ADNI as triangles.

To address this concern, we examined two longitudinal CSF data points for subjects who maintained normal cognition over 18 to 36 months. On the third clinical exam within 9 years of baseline, we identified decliners and non-decliners. The longitudinal CSF Aβ_42_ data revealed a polarization of Aβ_42_ levels for ε4 negative decliners (Fig 5). Specifically, the annual percentile Aβ_42_ ranking for non-carrier decliners moved to the extremes of the distribution showing either excessive increases or decreases in the longitudinal Aβ_42_ levels compared to the non-decliners. This effect was not observed in ε4 carriers. We also examined 18 subjects in the NYU cohort with three or more LP’s studied over 6–10 years. We did not observe for any subject evidence for CSF Aβ_42_ elevations followed by Aβ_42_ reductions. Presumably, the time course for AD is long and based on our cross-sectional observations, elevations are likely to have peaked years prior to the dementia transition period under surveillance. We conclude that the temporal course of tau and Aβ_42_ deposition in brain with respect to the CSF levels remains largely unknown and that longitudinal observations on younger subjects are needed to definitively establish that CSF Aβ_42_ level elevations occur in advance of cognitive decline, before CSF Aβ_42_ level decreases, and in advance of brain Aβ deposits.

It is worth briefly speculating that abnormal CSF clearance of CSF could affect the CSF analyte levels. It is increasingly apparent from animal studies that differential cross membrane transport properties, enzymatic degradation, molecular size, and brain glymphatic physiology contribute to the production and clearance of Aβ_42_ to the CSF [24]. While human studies have demonstrated reduced Aβ_42_ clearance to the CSF [25] and that reduced CSF Aβ_42_ levels are associated with brain Aβ depositions [1,2], it remains to be shown in humans that prior to brain deposition, CSF clearance is reduced and CSF Aβ_xx_ levels and X-Tau levels are elevated. Such reduced CSF clearance could potentially predict vulnerability to brain amyloid lesions and even tau aggregation. Recently, we reported using PET, a reduced CSF clearance in AD associated with elevated brain amyloid [26]. Others have shown this relationship in murine AD models [27]. Indirect evidence in support of a generalized clearance defect comes from our data showing strong linear relationships between X-Tau and both Aβ_38_ and Aβ_40_, as these Aβ fragments do not demonstrate appreciable brain depositions [22].

Second, we observed that over the 45 year age span examined, the mean CSF Aβ_42_ was not associated with age. Rather we observed an increased age associated variance that produced both high and low Aβ_42_ values. On the other hand, Aβ_40,_ Aβ_38,_ and X-tau were significantly and positively age associated. This result is consistent with one prior cross-sectional aging study that also examined a broad age range (47-84y) and similarly observed that the younger subjects (47-62y) showed a positive correlation between age and Aβ_42_ levels, whereas the older subjects showed a negative correlation [8]. Other studies that examined subjects of advanced age reported either no changes in Aβ_42_ [28,29] or reductions with age [30].

We offer that our broad age range, large sample size, and standardized clinical exams supports our explanation invoking a non-linear U-shaped quadratic function in the relationship of Aβxx with age and X-tau pathology. We observed a quadratic fit between Aβ_42_ and X-tau in each age quartile examined. However, the linear direction of the relationships changed from positive to negative with increasing age. Specifically, in the in the two younger age quartiles (between 45 and 70y), we observed a positive linear association between Aβ_42_ and tau and in the two older quartiles (between 70 and 90y) we observed that both the variance of Aβ_42_ increased and the slopes with age became negative. Overall, our results suggest that with increasing age, Aβ_42_ increases with X-tau up until a point where Aβ_42_ is either bound in plaques [11,31] or its activity dependent production is decreased [32]. However, as this effect is not found for other Aβ fragments which show consistent age dependent elevations, as others have concluded it points towards an Aβ_42_ deposition effect. We for the first time demonstrate in our longitudinal study that this nonlinear Aβ_42_ trajectory identifies subjects at risk for cognitive decline.

Third, our CSF biomarker predictions of clinical outcome only partially agree with prior findings of an increased risk of cognitive decline associated with elevations in the X-tau to Aβ_42_ ratio [33]. Our results demonstrate a new finding, an added risk associated with elevated CSF Aβ42 levels, the contribution of the Aβ^2^ term to the prediction. Specifically, Fagan et al first reported that only by combining baseline CSF P-tau181 and Aβ_42_ in a ratio were prediction effects seen for the transition from CDR = 0 to CDR >0.5 [31]. Similarly, others showed that a lower Aβ_42_ levels and higher P-tau181 levels best predicted future MCI/AD [34,35]. However, univariate CSF Aβ_42_ measures have not previously been shown to be predictive of cognitive decline. Consequently, our observation that a quadratic Aβ_42_ measure, one that considers both elevated and reduced levels as significant predictors is novel. This result is likely due to the added power of the large combined sample examining both younger and older decliners, and for the first time, the inclusion of Aβ_42_ elevations in the prediction models. We caution that the quadratic approach has not been applied to the transition between mild cognitive impairment and AD.

Overall, our results suggest that elevated tau is necessary for the prediction of cognitive decline across all age groups. However, in younger subjects considering Aβ_42_ elevations is very important as the cumulative evidence suggests Aβ_42_ elevations are followed by reductions. Future imaging studies are needed to examine the relationship between elevations in CSF Aβ_42_ and tau levels and brain depositions of these proteins. It is currently believed that CSF Aβ_42_ levels drop with brain aggregation but the antecedent relationship of elevations in the Aβ_42_ levels to brain deposition is unknown, but for one animal study [11]. Moreover, PET imaging data have yet to be reported for longitudinal CSF Tau level changes. Our CSF data are consistent with the view that CSF Tau and CSF Aβ_42_ elevations precede CSF Aβ_42_ reductions. This finding is not readily translatable to results from post mortem lesion staging that suggest an earlier tau deposition [6] but they do appear to conflict with formulations suggesting that initial CSF Aβ_42_ reductions drive the AD cascade in late onset AD [36].

In summary, we observed that quadratic relationships between Aβ_42_ and tau were consistently found across age quartiles in three independent normal aging cohorts. Most importantly, the quadratic (Aβ_42_)^2^ term improves on the linear Aβ_42_ in the prediction of future cognitive decline. The data suggest that the Aβ^2^ term affords an earlier recognition of risk, especially in younger subjects. Overall, our findings support the hypothesis that both high and low Aβ_42_ levels are associated with elevated Tau levels, and mark the earliest known risk stage for cognitive impairment related to preclinical AD. This observation may prove to be valuable in future secondary prevention trials. Further longitudinal imaging studies are needed to examine the non-linear time course of CSF Aβ_42_ and brain lesion deposition.
